# Relationships between indicators of prothrombotic activity and coronary microvascular dysfunction in patients with myocardial infarction with obstructive and non-obstructive coronary artery disease

**DOI:** 10.1186/s12872-022-02985-z

**Published:** 2022-12-06

**Authors:** Darya A. Vorobeva, Vyacheslav V. Ryabov, Julia G. Lugacheva, Konstantin V. Zavadovsky, Andrew V. Mochula

**Affiliations:** Tomsk National Research Medical Centre, Cardiology Research Institute, Russian Academy of Sciences, 111a, Kievskaya str, Tomsk, 634012 Russian Federation

**Keywords:** Myocardial infarction and non-obstructive coronary artery disease, Acute myocardial infarction, Myocardial perfusion imaging, Myocardial blood flow, Indicators of prothrombotic activity

## Abstract

**Abstract:**

The relationship between prothrombotic activity and coronary microvascular dysfunction (MVD) is limited. This study aimed to perform a comparative analysis of the relationship between prothrombotic activity and MVD in patients with myocardial infarction without obstructive coronary artery disease (MINOCA) and myocardial infarction with obstructive coronary artery disease (MI-CAD).

**Material and methods:**

A total of 37 patients were enrolled in the study; the main group included 16 MINOCA patients, and 21 MI-CAD patients were included in the control group. Blood samples for protein C, antithrombin, WF, plasminogen, and homocysteine were performed on the 4th ± 1 day of admission. CZT-SPECT data were used to determine the standard indices of myocardial perfusion dis-orders (SSS, SRS, and SDS), as well as stress and rest myocardial blood flow (MBF), myocardial flow reserve (MFR), and difference flows (DF). MVD was defined as MFR (≤ 1.91 ml/min); coronary slow flow (CSF) was defined as corrected TIMI frame count (21 ± 3).

**Results:**

We performed a step-by-step analysis of prothrombotic activity of the hemostasis system in binary logistic regression for MINOCA patients to identify factors associated with MVD (MFR ≤ 1.91 ml/min). A predictive model was developed to estimate the probability of reduced MFR. A low MFR is related to only plasminogen in MINOCA patients, whereas only wall motion score index (WMSI) in MI-CAD group was associated with a low MFR.

**Conclusion:**

This small-scale study revealed the relationship between indicators of prothrombotic activity and MVD. The key factors that affect MVD in MINOCA patients was plasminogen, whereas, in patients with MI-CAD, WMSI was the key factor. Measurements of MVD may enhance the risk stratification and facilitate future targeting of adjunctive antithrombotic therapies in MINOCA and MI-CAD patients.

## Introduction

Coronary artery thrombus is associated with the rupture or erosion of unstable atherosclerotic plaques and is the key mechanism for developing acute myocardial infarction (AMI) [1]. However, up to 15% of AMI patients have no evidence of obstructive coronary artery disease. AMI patients with non-obstructive coronary arteries (MINOCA) with heterogenous mechanisms for AMI development. [2, 3].

Despite the restored coronary arteries permeability in some patients, no complete restoration of perfusion can be observed in either patient with obstructive atherosclerosis (MI-CAD) or in patients free of that, which most likely indicates coronary microvascular dysfunction. Coronary microvasculature dysfunctions (MVD) occur in 16–20% of MI-CAD cases [2–4] and in 35–56% of MINOCA cases [5]. MVD increases the risk of adverse ischemic events in patients from both groups.

The state of coronary microvasculature cannot be assessed during invasive coronary angiography (ICA). Therefore, MVD is evaluated by functional assessment of coronary microcirculation, which can be performed by both invasive and non-invasive methods, including the TIMI flow grade or measurement of coronary flow reserve (CFR) by dynamic single-photon emission computed tomography (SPECT) [3, 6]. The latter approach allows simultaneous assessment of myocardial perfusion and CFR [3]. CFR is quantified as the ratio of maximal myocardial blood flow to the resting flow in epicardial arteries under pharmacological stress and at rest [7]. This indicator is a functional measure of large- and small-vessel ischemia.

It is known that MVD is associated with the development of heart failure with preserved ejection fraction, diabetes mellitus, hypertension, hypertrophic cardiomyopathy, and autoimmune diseases [8]. However, no data are available on the relationship of this pathology with thrombotic disorders that can be comorbid and aggravate MVD. It has been proven that perfusion disorders and MVD development are associated with atherothrombosis [3] in AMI and MICAD patients, and impaired vasodilation or vasoconstriction of coronary microvessels [9] in patients with MINOCA [5, 10, 11].

There are other reasons for MVD development in MINOCA patients: coronary thromboembolism or distal embolization and reperfusion injury of endothelial cells [3, 12]. In turn, distal embolization and coronary thromboembolism can occur with hereditary or acquired thrombotic disorders [13]. Screening for the presence of hereditary thrombophilia in MINOCA patients showed an incidence of about 14–33% [12, 14, 15], including factor V Leiden (− 12%), protein C and S deficiency, and factor XII deficiency (− 3%). A number of studies report the prevalence of other hereditary thrombophilia, such as hyperhomocysteinemia (HHC), increased levels of von Willebrand factor (VWF), and plasminogen deficiency, and their role in the development of thrombosis in patients with MINOCA [16, 17]. The prevalence of the acquired thrombotic disorders (antiphospholipid syndrome, myeloproliferative diseases) is about 20% [12, 14].

Despite the large bulk of data on the role of hereditary or acquired thrombotic disorders in patients with obstructive and non-obstructive atherosclerosis, there are no data on their relationship with MVD and prothrombotic activity. The relationship between prothrombotic activity and MVD may have important clinical implications for managing these patients and improving prognosis.

The objective of this study was to perform a comparative analysis of the relationship between prothrombotic activity and MVD in patients with MINOCA and MI-CAD.

## Material and methods

This study is a non-randomized, open, and controlled study. The study is registered on ClinicalTrials.gov: NCT03572023 (date of registration 28.06.2018) and contains the study protocol. The study was conducted according to the principles of the Declaration of Helsinki. The study was approved by the hospital Human Research Ethics Committee of the Research Institute of Cardiology, Tomsk National Research Medical Center, protocol No. 164 of 23-Nov-2017. All patients consistently admitted and included in the study between 2017 and 2018 provided voluntary informed consent.

The inclusion criteria for the MINOCA group were as follows: patients (18 years old and older) with ACS who underwent coronary angiography within 24 h after the onset of the disease, with no obstructive (≤ 50%) coronary atherosclerosis evidenced by the invasive coronary angiography results, high and moderate cardiovascular risk on the GRACE scale, and sinus rhythm on an electrocardiogram.

The exclusion criteria for the MINOCA group were: contraindication to adenosine administration, hemodynamic instability, myocardial inflammatory diseases, moderate-to-severe cardiac valvular disease, atrial fibrillation, previous revascularization, severe comorbidity, severe renal failure (eGFR < 30), pacing, and claustrophobia. Patients with Takotsubo syndrome were not included in this study.

The inclusion criteria for the MICAD group were patients (18 years old and older) with ACS who underwent ICA within 24 h after the disease onset, with stenosis ≥ 75% of one coronary artery, high and moderate cardiovascular risk on the GRACE scale, and sinus rhythm. The exclusion criterion for this group was myocardial infarction associated with revascularization; other criteria were similar to those for the MINOCA group.

### Baseline characteristics

**Fig. 1:**
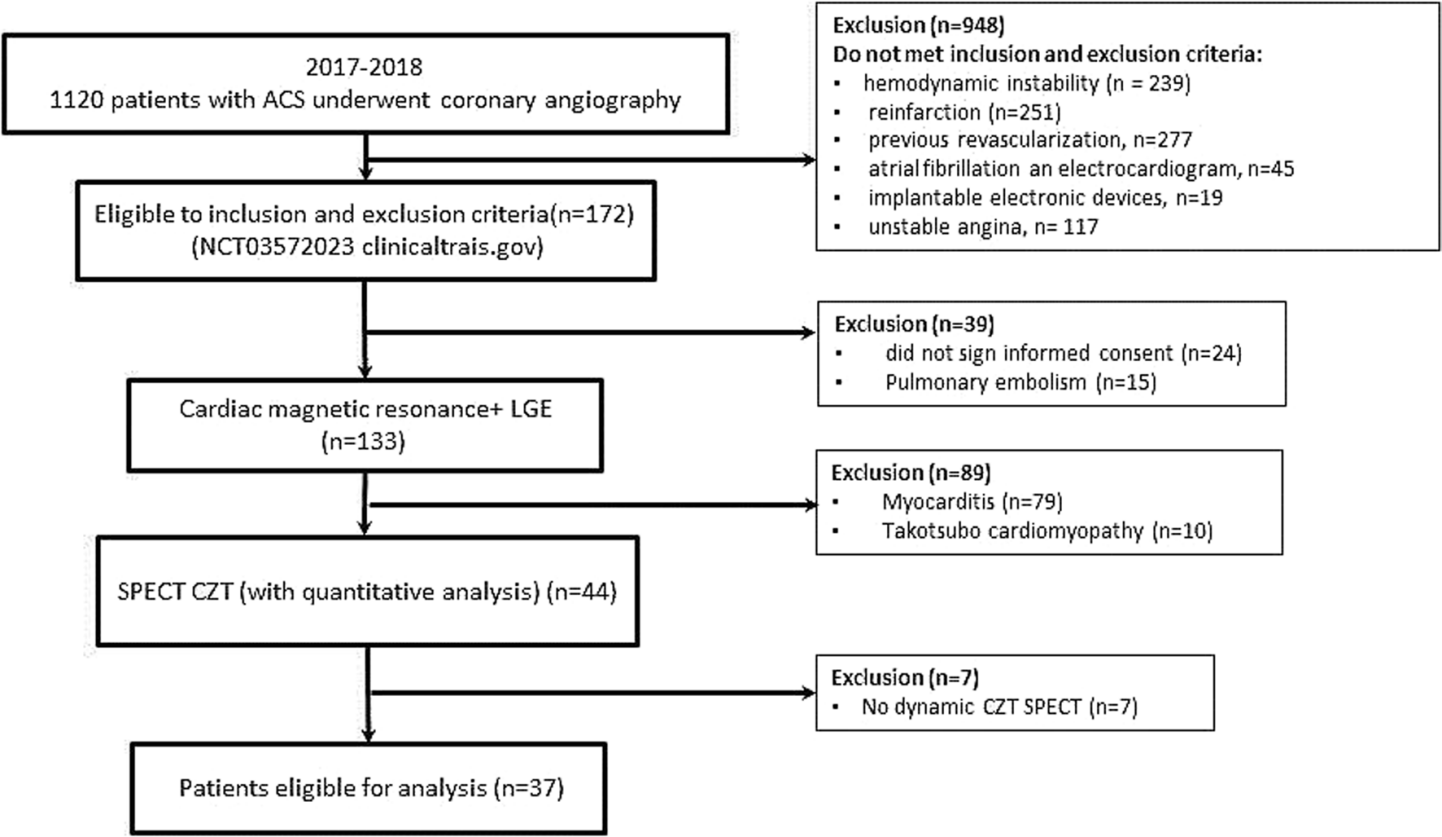
Flow diagram showing the patients included in the study. ACS, acute coronary syndrome. LGE, late gadolinium enhancement

The flow chart of the study is presented in Fig. 1. A total of 37 patients with myocardial infarction; 16 patients in the main group (MINOCA) and 21 patients served as the control group (MI-CAD) were enrolled in the study. Detailed clinical characteristics of the study group are presented in Table 1. After differential diagnosis, patients with acute myocarditis and Takotsubo cardiomyopathy confirmed by cardiac MRI were excluded from the study.

**Table 1:** Clinical, anamnestic characteristics and laboratory blood test results of patients

Number of patients, n%	MINOCA, n = 16	MICAD, n = 21	*p*-value
Men, n (%)	7 (43.7)	17 (80.9)	0.02
Age, Me (Q25; Q75)	66.0 (54.71)	60 (56;68)	0.33
Hypertension, n (%)	13 (81.3)	16 (76.1)	0.71
Dyslipidemia, n (%)	14 (87.5)	17 (80.9)	0.89
Overweight, n (%)	4 (25)	11 (52.3)	0.15
Family history of CAD, n (%)*	7 (43.7)	13 (61.9)	0.27
Smoking, n (%)	5 (31.3)	11 (52.3)	0.26
Diabetes mellitus, n (%)	0	4 (19.0)	0.02
GFR, ml / min / 1.73 m2, Me (Q25; Q75)	71.5 (54.0;80.0)	79.0 (65.0;89.0)	0.20
History of angina pectoris, n (%)	10 (62.5)	6 (28.5)	0.04
A history of stroke, n (%)	1 (5.2)	2 (9.5)	0.71
Peripheral atherosclerosis, n (%)	4 (25)	7 (33.3)	0.58
Time of admission to the hospital, min, Me (Q25; Q75)	390 (146.5;870)	180 (98;240)	0.02
STEMI, n (%)	10 (62.5)	19 (90.4)	0.01
GRACE, risk, Me (Q25; Q75)	2.0 (2.0;3.5)	2.3 (2.0;5.0)	0.26
Thrombolytic therapy, n (%)	3 (18.7)	11 (52.3)	0.007
TIMI 2 flow, n (%)	9 (56.3)	1(4.7)	0.01
Wall motion score index, score	1.0 (1.0;1.2)	1.2 (1.2;1.5)	0.04
Left ventricular ejection fraction, %	60.0 (45.0;60.0)	56.0 (50.0;60.0)	0.51
Acute apical left ventricle aneurysm with parietal thrombosis, n (%)	3 (18.7)	0	0.03
Acute apical left ventricle aneurysm, n (%)	0	2 (9.5)	0.62
Troponin I, ng/ml, 2 day	0.5 (0.11;8.3)	4.9 (1.0;25.2)	0.02
Troponin I, ng/ml, 4 day	0.4 (0.07;1.7)	0.7 (0.5;4.4)	0.04
Troponin I, ng/ml, 7 day	0.08 (0.02;0.2)	0.4 (0.2;0.9)	< 0.001

*Burdened heredity for cardiovascular pathology

*GFR* glomerular filtration rate; *STEMI* ST-segment elevation myocardial infarction

The differences in demographic, anamnestic characteristics and clinical characteristics were determined by gender, history of angina, time of hospital admission, the GRACE risk score, thrombolytic therapy at the prehospital stage, TIMI 2 flow detected by ICA, and the presence of acute left ventricle apical aneurysm with parietal thrombosis. The main clinical and anamnestic characteristics and laboratory blood data, such as levels of CPK-MB and troponins on days 2, 4, and 7 of hospitalization for MINOCA and MI-CAD patients, are summarized in Table 1.

Upon admission, all patients received standard therapy for acute coronary syndrome according to national recommendations; patients of both groups received dual antiplatelet therapy (100%), beta-blockers in 14 (87.5%) patients of the main group and 20 (95.2%) patients of the control group; angiotensin-converting enzyme inhibitors in 12 (75.0%) patients of the main group and 20 (95.2%) patients of the control group; calcium channel blockers were prescribed in seven (36.8%) patients of the main group and one (4.7%) patients of the control group; unfractionated heparin was obtained in 11 (68.7%) patients of the main group and 19 (90.4) patients of the control group, with subsequent transfer to low molecular weight heparins. After an additional examination, oral anticoagulants were administered to three (18.7%) patients of the main group and two (9.5%) patients of the control group.

### Invasive coronary angiography

All patients underwent quantitative coronary arteriography on an Axiom Artis coronary angiography system (Siemens; Erlangen, Germany. Coronary angiography in all patients was performed using a 5F Judkins-type catheter through the femoral approach. All coronary artery stenosis were quantitatively assessed using dedicated software by two experienced readers. Coronary artery stenosis ≥ 50% in major epicardial coronary arteries and in the left main coronary artery was considered significant. The criteria to define the coronary slow flow (CSF) is defined as a corrected frame count greater than 2 standard deviations from the normal range (21 ± 3) [18].

### Biochemical analysis

Hemostasiological and hematological blood tests were studied upon admission and on the 2nd, 4th, and 7th days of hospitalization. Blood samples for the study of troponin I were collected in a BD Vacutainer Gel and Clot Act. The upper limit of the norm was conventionally taken from the 99th percentile from the upper reference level (cTnI laboratory reference cut-off for normalcy: < 0.04 ng/ml). The content of these parameters in plasma was determined using an automatic hemostasis analyzer ACL TOP 700 (Instrumentation Laboratory Company, USA) with reagents produced by the same manufacturer. Blood samples for protein C, antithrombin, WF, plasminogen, and homocysteine were performed on the 4th ± 1 day of admission. Blood samples for protein C, antithrombin, WF, plasminogen, and homocysteine were collected in BD Vacutainer K3-EDTA. Blood samples to determine IgG/IgM antibodies to cardiolipin and β2-glycoprotein blood tests for lupus anticoagulant were collected in BD Vacutainer with 0.11 M (3.2%) sodium citrate after discontinuation of anticoagulant therapy. Blood samples for genetic analysis were collected in BD Vacutainer K3-EDTA.

The homocysteine level was determined by the enzyme immunoenzyme technique using AXIS-SHIELD 54 Diagnostics Limited (UK) set of instruments for diagnosis and standards methods. The samples’ optical density and standards were measured using a Sunrise microplate reader (TECAN, Austria) at the wavelength specified in the manufacturer’s instructions. The concentration of the determining factors was calculated using the Magellan software. To determine IgG/IgM antibodies to cardiolipin and β2-glycoprotein for diagnosing APS, the ORGENTEC Anti-β2-Glycoprotein I IgG/IgM ELISA enzyme immunoassay was used. Blood tests for lupus anticoagulants were performed using an ACL-Top 700 analyzer (Werfen) with HemosIL SynthASil dRVVT screen reagents/dRVVT confirm and with an SCT screen/SCT confirm quartz activator.

Chromogenic methods for determining the activity of antithrombin and protein C were employed to assess the anticoagulant potential of blood using an automatic hemostasis analyzer ACL TOP 700 (Instrumentation Laboratory Company, USA) and reagents produced by the manufacturer. The concentration of D-dimer in blood plasma was determined by the enzyme immunoassay using a reagent kit (TECHNOZYM Antigen EDTA ELISA, Technoclone, Austria). The samples’ optical density and standards were measured using a Sunrise microplate reader (TECAN, Austria) at the wavelength specified in the manufacturer’s instructions. The concentration of the determining factors was calculated using the Magellan software.

To assess fibrinolytic activity, the antigens of the tissue plasminogen activator (t-PA) and activator inhibitor type I (PAI-I) were determined by the enzyme immunoassay (TECHNOZYM Antigen EDTA ELISA, Technoclone, Austria). Plasminogen concentration in plasma was determined using an automatic hemostasis analyzer ACL TOP 700 (Instrumentation Laboratory Company, USA) and reagents produced by the same company.

Standard echocardiography was performed on the 4th day using a VIVID E9 ultrasound system (GE Healthcare). Within 2 weeks after admission, patients underwent dynamic CZT-SPECT MPI (with quantitative myocardial blood flow and myocardial flow reserve assessment). Afterward, both groups of patients performed per-patients and per-vessel analyses of semiquantitative and quantitative dynamic CZT-SPECT indices.

### Assessment of genetic polymorphisms

Heterozygous and homozygous variants of the following gene polymorphisms (distinguished from wildtype) were analyzed by eight polymorphic variants of the hemostatic system genes that were previously found to be associated with the risk of thrombophilia: factor II (FII), (20,210 G > A) rs1799963; factor V Leiden mutation (FV) (1691 G > A) rs6025; factor VII (FVII), (10976G > A) rs6046; factor XIII (F XIII), (163 G > T) rs5985; factor I (FI), (–455G > A) rs1800790; platelet receptor for collagen (GP Ia–IIa), (807C > T) rs1126643; platelet receptor for fibrinogen (GP IIb-IIIa), (1565 T > C) rs5918; plasminogen activator inhibitor type I (PAI-I) (–675 5G > 4G) rs1799889.

In addition, four polymorphic genotypes of folate cycle enzyme genes of included patients were analyzed: methylene-tetrahydro-folate-reductase (MTHFR), (677 C > T, 1298 A > C), methionine synthetase (MTR) (2756 A > G), methionine synthetase reductase (MTRR) (66 A > G).

Genotypes were determined using the polymerase chain reaction technique and a reagent kit produced by the company DNA-Technology. The study that employs reagent kits for detecting genetic polymorphisms involves the following stages: DNA extraction (sample preparation) and real-time PCR amplification. DNA was extracted using the PROBA-RAPID-GENETIKA kits (DNA-Technology, Russia) in compliance with the protocol proposed by the company. Add 100 µl peripheral blood (100 µl) was added to 600 µl lysis solution in sterile Eppendorf tubes and mixed by inverting the tubes. Saline (100 μl) was added to the negative control sample. The tubes were vortexed for 3–5 s and centrifuged at 13,000 rpm for 1 min. The supernatant was then removed. We then added 300 µl of Probe-46 Rapid reagent to the sediment and vortexed the tubes for 5–10 s. The tubes were then Incubated at 98 °C for 10 min before centrifuging at 13,000 rpm for 3 min. The prepared supernatant containing the extracted DNA was added to the reaction mixture for PCR amplification.

The PCR followed a conventional thermal cycling protocol using gene-specific primers and Taq polymerase. Signaling probes with fluorescent labels Fam and Hex were introduced into the mixture for amplification for each variant of the detected genetic polymorphism.

### Patient preparation

Patients were instructed to refrain from caffeine and methylxanthine-containing substances and to avoid nitrates, calcium channel blockers, and beta-blockers for at least 24 h before the scan. All scans were performed after overnight fasting.

### The dynamic CZT-SPECT acquisition protocol

All patients underwent rest-stress CZT (Discovery NM/CT 570c; GE Healthcare, Haifa, Israel) imaging in compliance with a two-day protocol. All patients were placed in the supine position. Before the first dynamic acquisition, a low-dose CT scan (tube voltage 120 kV, tube current 20 mA, rotation time 0.8 s, helical pitch 0.969:1, slice thickness 5 mm, and interstice interval of 5 mm) was performed for heart positioning and attenuation correction.

Three MBq/kg of 99mTc-Sestamibi were injected at rest using a syringe pump intravenously as a 5 ml bolus (injection rate 1 ml/sec) followed by saline flush (20 ml with the rate 2 ml/s, using an automatic injector Ulrich Missouri XD 2001 Ulrich GmbH & Co. KG, Ulm Germany). List mode ECG-gated dynamic data acquisition started 10 s prior to the radiopharmaceutical bolus injection and was acquired for 610 s. After 40 min from rest tracer injection, a 7 min long standard ECG-gated (16 framed per cardiac cycle) rest acquisition was performed using a dedicated patient positioning application in order to obtain the same coordinates of the heart as in the previous scan. The stress study was performed immediately after.

After 2 min of intravenous infusion of adenosine (160 mcg/kg/min), the second dose of 99mTc Sestamibi (9 MBq/kg) was injected, and list mode dynamic data acquisition of 610 s was started 10 s prior to the radiotracer injection. The infusion of adenosine continued for an additional 2 min [19]. After that, as for the rest scan, patients were removed from the gamma-camera, and a stress standard ECG-gated scan was acquired after 45 min from the tracer injection.

Low dose CT scans were transferred to the Xeleris workstation to obtain AC maps. The alignment of perfusion and CT data was done with visual control. CZT images were reconstructed on the dedicated workstation (Xeleris 4.0; GE Healthcare, Haifa, Israel) using maximum-penalized-likelihood iterative reconstruction (60 iterations; Green OSL Alpha 0.7; Green OSL Beta 0.3) to acquire perfusion images in standard cardiac axes (short axis, vertical long axis, and horizontal long axis). The software Myovation for Alcyone (GE Healthcare, Haifa, Israel) was used for image reconstruction, and the Butterworth post-processing filter (frequency 0.37; order 7) was applied to the reconstructed slices. The reconstruction was performed in a 70 × 70 pixels matrix with 50 slices.

Each of 17 segments was scored based on semiquantitative 5-point scoring system (from 0 = normal uptake to 4 = absent radiotracer distribution) [20]. Accordingly, the sum of the stress scores of all segments (SSS) and the sum of the rest scores of all segments (SRS) was quantified. A summed difference score (SDS) was calculated as the difference between SSS and SRS. Image processing was performed at the Core Facility “Medical Genomics” (Tomsk National Research Medical Center, Tomsk, Russia). Dynamic CZT imaging was processed as previously published [21]. To convert the tracer uptake rate to MBF values, the Renkin–Crone flow model was used with the following parameters: a = 0.879, b = 0.337 for attenuation corrected images [22]. The value of MFR was calculated as the MBF ratio (MBF stress/MBF rest). Additionally, the absolute difference between stress MBF and rest MBF as flow difference (FD) was calculated [22, 23]. Myocardial blood flow and coronary flow reserve were determined after 7–10 days from the onset of the AMI. CZT-SPECT data were used to determine the standard indices of myocardial perfusion disorders (SSS, SRS, and SDS), as well as stress and rest myocardial blood flow (MBF), myocardial flow reserve (MFR), and difference flows (DF) [21]. The predictor of MVD is the value MFR ≤ 1.91 [21].

#### Statistical analysis

Statistical analyses were performed using the STATISTICA 10, StatTech v. 2.8.8 (Developer—StatTech LLC, Russia). The distribution of continuous variables was checked by using the Shapiro–Wilk W-test. Continuous variables were expressed as mean ± standard deviation and median with quartiles (Q25–Q75). For qualitative indicators, n (%) was taken, where n is an absolute number and % is a relative value in percent. Nominal data were analyzed using the Pearson *χ*2 test and two-sided Fisher’s exact test (at expected frequencies less than 5). The level of significance was adjusted for multiple testing using Bonferroni error correction. The Spearman test was used to estimate the correlation coefficient between quantitative variables. The studied values did not agree with the normal law; therefore, the nonparametric Mann–Whitney test was used to assess the differences in independent samples. The development of a prognostic model for the probability of a binary outcome was carried out using logistic regression. Accuracy of MVD detection was assessed by receiver operator characteristic (ROC) analysis, reporting areas under the curve (AUC), and their associated 95% confidence intervals. The best values in the prediction of MVD (MFR ≤ 1.91) were defined as the cut-off point having the highest Youden Index. The *p*˂0.05 value was considered statistically significant.

## Results

### Prothrombotic state and polymorphic variants of genes of the hemostasis system

The study of polymorphic variants of genes of the hemostasis system and platelet receptors that can cause thrombosis revealed unfavorable homozygous variants for genes of factor XIII, factor I, and PAI-I in 12 (75.0%) patients of the main group. In the control group, unfavorable homozygous gene variants were detected in 19 (90.4%) patients concerning homozygous variants similar to those recorded in the main group. In contrast to the control group, patients of the main group were statistically less likely to have a heterozygous factor XIII genotype (*p* = 0.04). No statistical changes were found for other studied gene polymorphisms (*p* > 0.05).

The analysis of allelic variants of genes of the folate cycle enzymes revealed that 13 (81.3%) patients of the main group had homozygous variants for genes: MTHFR (677C > T), MTRR (66A > G), MTHFR (1298 A > C), and MTR (2756 A > G). Among patients with MI-CAD, 11 (52.4%) patients had homozygous variants for genes: MTRR (66A > G), MTHFR (1298 A > C), and MTR (2756 A > G). The analysis showed no statistical differences in those genes encoding the folate cycle enzymes (*p* > 0.05).

The prothrombotic activity data indicated that patients with MINOCA had a lower level of plasminogen (*p* = 0.007) and a higher level of homocysteine (*p* = 0.03). No differences between the two groups were found in terms of protein C, antithrombin, and WF (all *p* > 0.05). At the same time, protein C deficiency was detected in two (12.5%) MINOCA patients and two 2 (9.5) MI-CAD patients. Antithrombin deficiency was recorded in two (12.5%) MINOCA patients. Plasminogen deficiency was recorded in three (18.8%) MINOCA patients and one (4.8%) MI-CAD patient. An increased homocysteine level was found in nine (56.3%) MINOCA patients and six (28.6%) MI-CAD patients. An increased WF level was found in six (37.5%) MINOCA patients and seven (33.3%) MI-CAD patients. No differences were found in the presence of lupus anticoagulants and antibodies to cardiolipin and β2-glycoprotein (*p* > 0.05). Indicators of the prothrombotic state are summarized in Table 2.

**Table 2:** Indicators of the prothrombotic state

Indicator (reference range)	MINOCA, n = 16	MICAD, n = 21	*p*-value
Protein C, % (70–130)	111.6 (101.9;120.1)	127 (122.3;132,2)	0.36
Protein C deficiency, < 70%	2(12.5)	2 (9.5)	0.91
Antithrombin, % (83–128)	101.6 (96.6;110.2)	124 (119.0;129.2)	0.86
Antithrombin deficiency, n%	2 (12,5)	0	0.10
Willebrand factor, % (69–116)	106.5 (90.0;116.0)	117 (114.3;118.7)	0.35
Willebrand factor deficiency, n%	6 (37.5)	7 (33.3)	0.10
Homocysteine, mcmol/L (4,1–10,2)	12.1 (11.0;14.0)	10.4 (8.7;12.5)	0.03
Hyperhomocysteinemia, n%	9 (56.3)	5 (23.8)	0.04
Plasminogen, % (80–135)	113.1 (101.3;119.8)	122.0 (115.2;127.1)	0.007
Plasminogen deficiency, n%	3 (18.8)	1 (4.8)	0.23
IgG antibodies to cardiolipin, n%	0	0	–
IgM antibodies to cardiolipin, n%	2 (25)	2 (9.5)	0.62
IgG antibodies to β2-glycoprotein, n%	0	0	–
IgM antibodies to β2-glycoprotein-I, n%	2 (25)	2 (9.5)	0.65
Lupus anticoagulant, n%	9 (56.2)	11(52.3)	0.62

### Dynamic CZT-SPECT results

The myocardial perfusion data recorded at rest and after a pharmacological stress test showed that myocardial perfusion and myocardial blood flow indicators under pharmacological stress in MI-CAD patients are lower than those in MINOCA patients. No differences were found for other indicators. The proportion of patients with decreased MBF and MFR is comparable in both groups. Indicators of myocardial perfusion in patients of both groups are summarized in Table 3.

**Table 3:** Dynamic CZT-SPECT results

Indicator	MINOCA, n = 16	MICAD, n = 21	p-value
SSS, score	5.0 (3.0;6.5)	9.0 (5.0; 13.0)	0.01
SRS, score	3.0 (1.5;4.0)	4.0 (3.0;11.0)	0.13
SDS, score	2.0 (1.0;3.0)	3.0 (2.0;5.0)	0.02
MBF stress, ml/min/g	1.2 (0.95;1.6)	0.8 (0.6;1.1)	0.002
MBF rest, ml/min/g	0.8 (0.4;1.1)	0.5 (0.3;0.8)	0.04
MFR, ml/min/g	1.9 (1.2;2.2)	1.3(1.1;2.1)	0.3
Reduced MFR total, n (%)	9 (56.2)	11 (52.3)	0.37
DF total, ml/min/g	0.5 (0.1;0.8)	0.1 (0.02;0.5)	0.16

*SSS* summed stress score; *SRS* summed rest score; *SDS* summed difference score (SDS = SSS − SRS); *MBF* myocardial blood flow, *MFR* myocardial flow reserve; *DF* difference flows

### Relationship between indicators of prothrombotic activity, instrumental data and myocardial flow reserve

The data obtained for the MINOCA patients revealed a correlation between SSS and troponin I on day 4 (r = 0.56, *p* = 0.02) and day 7 (r = 0.79, *p* = 0.0002); SRS and troponin I on day 2 (r = 0.58, *p* = 0.02), on day 4 (r = 0.59, *p* = 0.02), and on day 7 (r = 0.65, *p* = 0.01); SSS and WMSI (r = 0.68, *p* = 0.003); SRS and WMSI (r = 0.61, *p* = 0.01).

In addition, a negative correlation was revealed between MFR and troponin I on day 2 (r =  − 0.54, *p* = 0.006) and on day 4 (r =  − 0.50, *p* = 0.002); MFR and WMSI (r =  − 0.40, *p* = 0.04), MFR and antithrombin (r =  − 0.48, *p* = 0.005); a moderate correlation between MFR and plasminogen (r = 0.61, *p* = 0.01), between the serum level of protein C and AT (r = 0.65, *p* = 0.0001), AT and WF (r = 0.54, *p* = 0.0001); a negative correlation between homocysteine and plasminogen (r =  − 0.69, *p* = 0.002).

In patients with MI-CAD, a correlation between SRS and troponin I was revealed on day 2 (r = 0.44, *p* = 0.04); SSS and WMSI (r = 0.53, *p* = 0.01), SRS and WMSI (r = 0.49, *p* = 0.03); MFR and troponin I on day 7 (r =  − 0.41, *p* = 0.02); MFR and WMSI (r =  − 0.60, *p* = 0.03).

Logistic regression analysis was used to determine factors that influence the development of MVD. We performed step-by-step inclusion of prothrombotic activity, laboratory and instrumental data in the analysis of binary logistic regression for patients of the MINOCA group to identify factors associated with reduced MFR (≤ 1.91 ml/min). A predictive model was developed to estimate the probability of MFR. The analysis revealed that reduced MFR is related to only plasminogen. The observed association can be described by the following equation:

$$\begin{gathered} {\text{MVD }} = \, 1 \, / \, \left( {1 \, + {\text{ e}} - {\text{z}}} \right) \, \times \, 100\% \hfill \\ {\text{z }} = \, - 1.630 \, + \, 0.584 \times {\text{ plasminogen}} \hfill \\ \end{gathered}$$

**Fig. 2:**
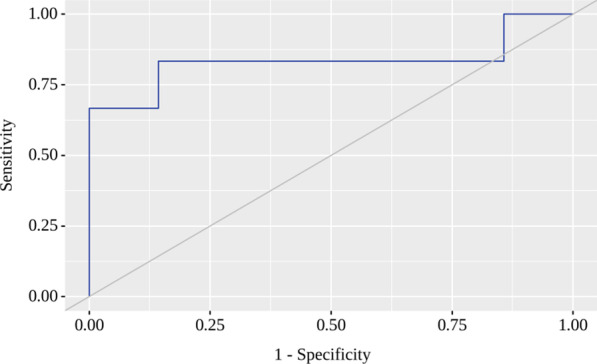
ROC-curve characterizes the probability of MVD’s dependence on the plasminogen

The resulting regression model is statistically significant (*p* = 0.008). Based on the signs of the regression coefficients, a direct relationship of plasminogen with MVD probability was established. The area under the ROC curve comprised 0.833 ± 0.084 with 95% CI 0.669–0.997 (Fig. 2). The resulting model was statistically significant (*p* = 0.004). The sensitivity and specificity of the method were 83.3% and 85.7%, respectively. Characteristics of the association of predictors with the probability of MVD are presented in Table 4.

**Table 4:** Characteristics of the association of predictors with the probability of MVD

Predictors	Unadjusted		Adjusted	
	COR; 95% CI	*p*	AOR; 95% CI	*p*
Plasminogen	1.019; 0.961–1.081	0.04*	1.108; 1.006–1.221	0.03*

*Association of the outcome value with the predictor value is statistically significant (*p* < 0.05)

Thus, we performed step-by-step inclusion of prothrombotic activity and laboratory and instrumental data in the analysis of binary logistic regression for the MI-CAD group to identify factors associated with reduced MFR. A low MFR is related to only WMSI in MI-CAD patients. The observed association can be described by the following equation:

$$\begin{gathered} {\text{MVD}} = 1/\left( {1 + {\text{e}} - {\text{z}}} \right)\; \times \;100\% \hfill \\ {\text{z}} = - 7.705 \, + \, 7.065\; \times \;{\text{WMSI}} \hfill \\ \end{gathered}$$

**Fig. 3:**
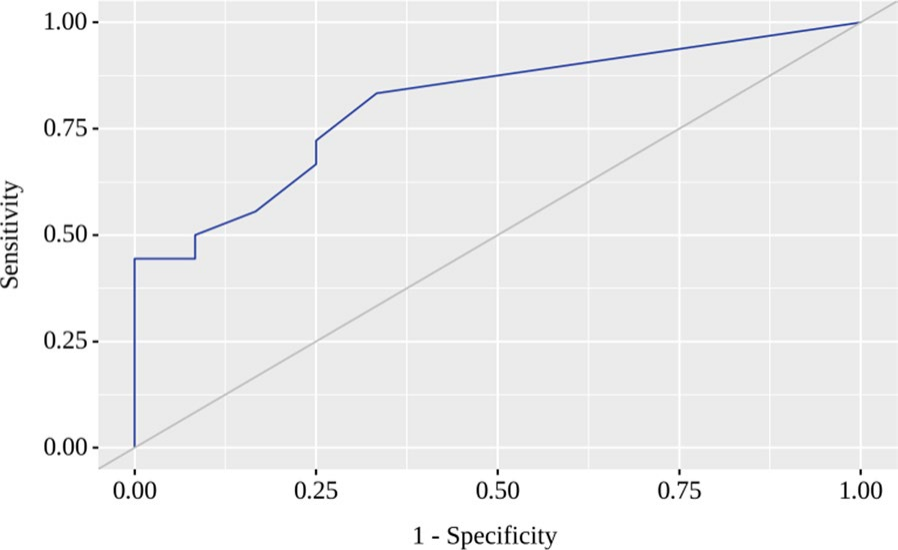
ROC-curve characterizes the dependence of the probability MVD on the WMSI value

The resulting regression model is statistically significant (*p* < 0.001). Based on the signs of the regression coefficients, a direct relationship of WMSI with MVD probability was established. The area under the ROC curve comprised 0.808 ± 0.055 with 95% CI 0.700–0.916 (Fig. 3). The resulting model was statistically significant (*p* < 0.001). The sensitivity and specificity of the method were 83.3% and 66.7%, respectively. Characteristics of the association of predictors with the probability of MVD are presented in Table 5.

**Table 5:** Characteristics of the association of predictors with the probability of MVD

Predictors	Unadjusted		Adjusted	
	COR; 95% CI	*p*	AOR; 95% CI	*p*
WMSI	1169.814; 9.450–144,784.253	0.004*	1169.814; 9.450–144,784.253	0.004*

*Association of the outcome value with the predictor value is statistically significant (*p* < 0.05)

*WMSI* wall motion score index of left ventricular

## Discussion

This is the first study to determine the relationship between indicators of prothrombotic activity and MVD in patients with MINOCA and MI-CAD. In the study, thrombotic parameters are considered only as some of the mechanisms of MVD. Although other disorders, such as microvascular spasm and microvascular obstruction, can play some role [5], they have not been considered in the study.

This study did not reveal any difference between carriage of polymorphic variants of genes of the hemostasis system and folate cycle enzymes in patients of both groups, which was in line with the data reported in previous study [12, 15]. Patients with MINOCA showed greater prothrombotic activity compared to patients with MI-CAD due to a greater increase in homocysteine and plasminogen deficiency. These results exceed those obtained by Pasupathy S, et al. and Da Costa A, et al., where no differences between the groups were found [15, 24]. The discrepancies between these data can be attributed to differences in the prevalence among the population depending on its ethnicity, differences in inclusion criteria, variations in dosing methods or depending on the sampling time. However, no antithrombin was detected in all these studies of differences in the level of protein C deficiency.

Indicators of prothrombotic activity contribute to the development of the thrombotic state [25, 26]. The state of thrombotic readiness is critical for developing perfusion disorders and MVD in patients with AMI [27].

Moreover, according to other results, MINOCA patients showed lower troponin I, WMSI, and abnormal myocardial perfusion indices. This suggests less myocardial injury in patients with MINOCA compared to patients with MI-CAD, whereas the indicators of myocardial blood flow (MBF stress, MBF rest) were lower in MI-CAD patients. Thus, the larger the area of myocardial injury, the greater the decrease in MBF and MFR [28], as demonstrated by our team previously [29].

Impaired myocardial perfusion does not always involve MVD in the early post-infarction period. According to the consensus of experts on microvascular angina, MFR values below 2.0–2.5 indicate microvascular dysfunction [8, 30]. However, these values are calculated for patients with stable coronary artery disease. In the present study, only 50% of patients showed signs of decreased MFR according to the reference values for AMI patients.

It is known that ischemic events precede microvascular dysfunction. However, a novel concept of the mechanisms resulting in acute coronary syndromes takes into account the primary dysfunction of the microcirculation as one more factor for myocardial injury [30]. It cannot be unambiguously concluded that the development of microvascular dysfunction in AMI patients is due only to ischemia since there are no initial values of the coronary blood flow. In this case, MBF and MFR are integrated indicators of the response to the decreased blood flow resulting from ischemia, myocardial reperfusion, and MVD [8, 31].

Patients with CSF were found to have elevated levels of HHC associated with endothelial dysfunction, which may contribute to the development of AMI development in patients with MINOCA [32–35]. During HHC, homocysteine, and plasminogen compete for receptors of the cofactor annexin. The conversion of plasminogen to plasmin is disrupted, fibrinolytic activity decreases, and the risk of thrombosis increases [36]. At the same time, the negative relationship between homocysteine and plasminogen confirms the effect of HHC on a decrease in fibrinolytic activity and an increase in the risk of thrombus formation in patients of the main group [33, 34].

We included indicators that influenced myocardial perfusion in the logistic analysis and found that only the level of plasminogen affected the development of MVD in MINOCA patients. The effect of homocysteine and antithrombin on MVD in MINOCA patients was not found due to a small sample of patients, but it cannot be ruled out. With an increase in the sample of patients, the effect of homocysteine and antithrombin will be more pronounced.

In the MI-CAD group, only the WMSI value affected the development of MVD. These data suggest a different genesis of the development of MVD due to myocardial infarction and indicators of prothrombotic activity in both groups.

The most common microvascular causes of MINOCA are microvascular spasm and the obliteration of coronary microcirculation linked to thrombophilic disorders or thromboembolism [9]. Chang et al. evaluated the influence of thrombogenicity on MVD occurrence and its prognostic implications in MINOCA patients, but they did not study indicators of prothrombotic activity in these patients. Chang et al. [37] concluded that high platelet–fibrin clot strength is associated with decreased coronary flow reserve and worse prognosis.

Relationship between prothrombotic activity and MVD has a major clinical implication for managing these patients and improving prognosis. Our findings can provide novel strategies for the prevention of MVD in patients with MINOCA. Further exploration could improve understanding and provide new treatments for patients with MINOCA.

More intensified antithrombotic therapy, such as a glycoprotein IIb/IIIa inhibitor after ICA or new oral anticoagulants for long-term treatment, can be considered for MINOCA patients with high prothrombotic activity. The results obtained require further investigation and confirmation in a larger sample of patients.

## Conclusion

This small-scale study revealed the relationship between indicators of prothrombotic activity and MVD. The key factor that affected MVD in MINOCA patients was plasminogen. In patients with MI-CAD, WMSI was the key factor associated with MVD. Measurements of MVD may enhance the risk stratification and facilitate future targeting of adjunctive antithrombotic therapies in patients with obstructive and non-obstructive coronary artery disease.

## Limitations

This study was conducted as a single-center trial. A small sample of patients was involved in the study due to more stringent inclusion criteria and time constraints to conduct the pilot study. Results may not be generalizable to populations of different ethnic backgrounds, and the definition of MVD was based on the MFR-derived CZT-SPECT. Further studies are needed to confirm the findings with the golden standard of MVD assessment, e.g., the invasive index of microcirculatory resistance or coronary flow reserve. Since MINOCA patients are characterized by mild reduction of myocardial blood flow and perfusion assessed visually and quantitatively, this group of patients is likely to have a more pronounced risk of cardiac events and needs more aggressive observation and treatment, despite the absence of obstructive coronary artery lesion. It requires further large-scale studies to test the prognostic significance of SPECT-derived MBF and MFR in patients with acute coronary syndrome.

## Data Availability

The datasets used and/or analysed during the current study available from D. Vorobeva on reasonable request.

## Abbreviations

AMI: Acute myocardial infarction
MINOCA: Myocardial infarction and non-obstructive coronary artery disease
MICAD: Myocardial infarction and obstructive atherosclerosis
CFR: Coronary flow reserve
SPECT: Single-photon emission computed tomography
HHC: Hyperhomocysteinemia
VWF: Von Willebrand factor
ICA: Invasive coronary angiography
CSF: Coronary slow flow
WMSI: Wall motion score index
MBF: Myocardial blood flow
MFR: Myocardial flow reserve
MVD: Microvascular disfunction
DF: Difference flows
